# Supplementary material for: Extracellular vesicles therapy alleviates cisplatin-ınduced testicular tissue toxicity in a rat model
Source: PLoS One. 2025 May 2;20(5):e0314093. doi: 10.1371/journal.pone.0314093 (PMC12047789; doi:10.1371/journal.pone.0314093)
Supplement: Attachment — Submitted filename: PONE-D-24-32691_reviewer.pdf [file pone.0314093.s001.pdf]

PLOS ONE

EVs Therapy for Cisplatin-Induced Testicular Tissue Toxicity

--Manuscript Draft--

**Manuscript Number:**	PONE-D-24-32691
**Article Type:**	Research Article
**Full Title:**	EVs Therapy for Cisplatin-Induced Testicular Tissue Toxicity
**Short Title:**	EVs in Mitigating Cisplatin-Induced Testicular Toxicity
**Corresponding Author:**	HALIME TOZAK YILDIZ, Ph.D.<br>Kirsehir Ahi Evran Universitesi<br>Kırşehir, Merkez TÜRKIYE
**Keywords:**	extracellular vesicles; mesenchymal stem cells; cisplatin; testicular damage; autophagy
**Abstract:**	Purpose: Cisplatin, a chemotherapy drug for various cancers, causes significant infertility and gonadotoxicity due to its metabolites. Testicular dysfunction from cisplatin is often a result of oxidative stress and autophagy. This study investigates the regenerative, antioxidant, and autophagy-regulating properties of extracellular vesicles to mitigate cisplatin-induced testicular damage.<br>Methods: Cisplatin toxicity was induced with a 7.5mg/kg dose on day one. On day six, a single dose of extracellular vesicles (80x106/100 µl) was administered. Tissue samples were collected on day eight for analysis. Testicular morphology was examined using hematoxylin and eosin staining. Sperm motility and count were assessed. Immunohistochemistry was used to evaluate autophagy markers, SF-1, and StAR expressions. Oxidative stress markers, inhibin, and testosterone levels were measured.<br>Results: Cisplatin-induced damage included a reduction in germinal epithelium, interstitial edema, and degeneration in seminiferous tubules, along with a decline in sperm motility and quality. An increase in the expression of autophagy markers, including Beclin-1, p62, LC3-2, SF-1, and StAR, was observed, along with elevated MDA activity and a reduction in GSH-PX, SOD, and CAT activities. Testosterone and inhibin levels also declined. In the extracellular vesicles-treated group, tissue morphology was restored, autophagy marker expression was reduced, and SF-1 and StAR expressions were increased. Oxidative stress markers decreased, and hormone levels improved.<br>Conclusion: Extracellular vesicles show promise in reducing cisplatin-induced reproductive toxicity and testicular damage by modulating autophagy and oxidative stress, suggesting a potential therapeutic approach for preserving fertility in patients undergoing cisplatin chemotherapy.
**Order of Authors:**	Halime TOZAK YILDIZ, Ph.D.
	Kübra Tuğçe KALKAN, PhD
	Numan BAYDİLLİ, MD
	Zeynep Burçin GÖNEN
	Özge CENGİZ MAT
	Eda KÖSEOĞLU
	Gözde Özge ÖNDER
	Arzu YAY, PhD
**Additional Information:**	
**Question**	**Response**
**Financial Disclosure**<br><br>Enter a financial disclosure statement that	The author(s) received no specific funding for this work.; The author(s) received no specific funding for this work.

Powered by Editorial Manager® and ProduXion Manager® from Aries Systems Corporation

describes the sources of funding for the work included in this submission. Review the submission guidelines for detailed requirements. View published research articles from *PLOS ONE* for specific examples.<br><br>This statement is required for submission and **will appear in the published article** if the submission is accepted. Please make sure it is accurate.<br><br>**Funded studies**<br>Enter a statement with the following details:<br>• Initials of the authors who received each award<br>• Grant numbers awarded to each author<br>• The full name of each funder<br>• URL of each funder website<br>• Did the sponsors or funders play any role in the study design, data collection and analysis, decision to publish, or preparation of the manuscript?<br><br>Did you receive funding for this work?	
**Competing Interests**<br><br>Use the instructions below to enter a competing interest statement for this submission. On behalf of all authors, disclose any competing interests that could be perceived to bias this work—acknowledging all financial support and any other relevant financial or non-financial competing interests.<br><br>This statement is required for submission and **will appear in the published article** if the submission is accepted. Please make sure it is accurate and that any funding sources listed in your Funding Information later in the submission form are also declared in your Financial Disclosure statement.<br><br>View published research articles from *PLOS ONE* for specific examples.	The authors have declared that no competing interests exist.The authors have declared that no competing interests exist.

Powered by Editorial Manager® and ProduXion Manager® from Aries Systems Corporation

**NO authors have competing interests**<br><br>Enter: *The authors have declared that no competing interests exist.*<br><br>**Authors with competing interests**<br><br>Enter competing interest details beginning with this statement:<br><br>*I have read the journal's policy and the authors of this manuscript have the following competing interests: [insert competing interests here]*<br><br>* typeset	
**Ethics Statement**<br><br>Enter an ethics statement for this submission. This statement is required if the study involved:<br><br>• Human participants<br>• Human specimens or tissue<br>• Vertebrate animals or cephalopods<br>• Vertebrate embryos or tissues<br>• Field research<br><br>Write "N/A" if the submission does not require an ethics statement.<br><br>General guidance is provided below. Consult the submission guidelines for detailed instructions. **Make sure that all information entered here is included in the Methods section of the manuscript.**	The experiments were conducted in accordance with The ARRIVE guidelines (Animal Research: Reporting of In Vivo Experiments). All experimental protocols were approved by the Local Ethic Committee of Animal Experiments of Erciyes University (approval no. 22/185)in Turkiye. Rats in the groups were sacrificed by cervical dislocation after testicular and blood samples were taken under xylazine (10 mg/kg, IP) and ketamine (60 mg/kg, IP) general anesthesia.Experiments were carried out following the Declaration of Helsinki and the National Institutes of Health's (USA) Guide for the Care and Use of Laboratory Animals. All experimental protocols were approved by the Local Ethic Committee of Animal Experiments of Erciyes University (approval no. 22/185)in Turkiye. Rats in the groups were sacrificed by cervical dislocation after testicular and blood samples were taken under xylazine (10 mg/kg, IP) and ketamine (60 mg/kg, IP) general anesthesia.

Powered by Editorial Manager® and ProduXion Manager® from Aries Systems Corporation

**Format for specific study types**<br><br>**Human Subject Research (involving human participants and/or tissue)**<br>• Give the name of the institutional review board or ethics committee that approved the study<br>• Include the approval number and/or a statement indicating approval of this research<br>• Indicate the form of consent obtained (written/oral) or the reason that consent was not obtained (e.g. the data were analyzed anonymously)<br><br>**Animal Research (involving vertebrate animals, embryos or tissues)**<br>• Provide the name of the Institutional Animal Care and Use Committee (IACUC) or other relevant ethics board that reviewed the study protocol, and indicate whether they approved this research or granted a formal waiver of ethical approval<br>• Include an approval number if one was obtained<br>• If the study involved *non-human primates*, add *additional details* about animal welfare and steps taken to ameliorate suffering<br>• If anesthesia, euthanasia, or any kind of animal sacrifice is part of the study, include briefly which substances and/or methods were applied<br><br>**Field Research**<br><br>Include the following details if this study involves the collection of plant, animal, or other materials from a natural setting:<br>• Field permit number<br>• Name of the institution or relevant body that granted permission	
**Data Availability**<br><br>Authors are required to make all data underlying the findings described fully available, without restriction, and from the time of publication. PLOS allows rare exceptions to address legal and ethical concerns. See the PLOS Data Policy and FAQ for detailed information.	Yes - all data are fully available without restriction; Yes - all data are fully available without restriction

A Data Availability Statement describing where the data can be found is required at submission. Your answers to this question constitute the Data Availability Statement and **will be published in the article**, if accepted.<br><br>**Important:** Stating 'data available on request from the author' is not sufficient. If your data are only available upon request, select 'No' for the first question and explain your exceptional situation in the text box.<br><br>Do the authors confirm that all data underlying the findings described in their manuscript are fully available without restriction?	
**Describe where the data may be found in full sentences. If you are copying our sample text, replace any instances of XXX with the appropriate details.**<br><br>• If the data are **held or will be held in a public repository**, include URLs, accession numbers or DOIs. If this information will only be available after acceptance, indicate this by ticking the box below. For example: *All XXX files are available from the XXX database (accession number(s) XXX, XXX.).*<br>• If the data are all contained **within the manuscript and/or Supporting Information files**, enter the following: *All relevant data are within the manuscript and its Supporting Information files.*<br>• If neither of these applies but you are able to provide **details of access elsewhere**, with or without limitations, please do so. For example:<br><br>*Data cannot be shared publicly because of [XXX]. Data are available from the XXX Institutional Data Access / Ethics Committee (contact via XXX) for researchers who meet the criteria for access to confidential data.*<br><br>*The data underlying the results presented in the study are available from (include the name of the third party*	All relevant data are within the manuscript and its Supporting Information files.All relevant data are within the manuscript and its Supporting Information files.

and contact information or URL).<br>• This text is appropriate if the data are owned by a third party and authors do not have permission to share the data.<br><br>* typeset	
Additional data availability information:	Tick here if the URLs/accession numbers/DOIs will be available only after acceptance of the manuscript for publication so that we can ensure their inclusion before publication.; Tick here if the URLs/accession numbers/DOIs will be available only after acceptance of the manuscript for publication so that we can ensure their inclusion before publication.

Manuscript inc. figure
Click here to access/download;Manuscript;02.08.2024 Manuscript inc.figure .docx

TİTLE PAGE

Article Title: EVs Therapy for Cisplatin-Induced Testicular Tissue Toxicity

Halime TOZAK YILDIZ PhD [a], Kübra Tuğçe KALKAN Ph.D.[a], Numan BAYDİLLİ MD [b], Zeynep Burçin GÖNEN PhD[c], Özge CENGİZ MAT Asist[d], Eda KÖSEOĞLU Asist[d], Gözde Özge ÖNDER PhD[d], Arzu YAY Prof[d]

a Department of Histology and Embryology, Faculty of Medicine, Kirsehir Ahi Evran University, 40100 Kirsehir, Turkey Tel: +903862803900

b Department of Urology, Faculty of Medicine, Erciyes University, Melikgazi, 38030, Kayseri, Turkey Tel: +903524374910

c Betül-Ziya Eren Genome and Stem Cell Center (GENKOK), Erciyes University, Melikgazi, 38030, Kayseri, Turkey   Tel: +903524375272

d Department of Histology and Embryology, Faculty of Medicine, Erciyes University, Melikgazi, 38030, Kayseri, Turkey Tel: +903524374910

Corresponding Author: Halime TOZAK YILDIZ

Department of Histology and Embryology, Faculty of Medicine, Kirsehir Ahi Evran University, 40100 Kirsehir, Turkey

E-mail adress: htyildiz@ahievran.edu.tr, hhalimeyildiz@hotmail.com

Tel: +90 386 280 39 00- 2546

Abstract

Purpose: Cisplatin, a chemotherapy drug for various cancers, causes significant infertility and gonadotoxicity due to its metabolites. Testicular dysfunction from cisplatin is often a result of oxidative stress and autophagy. This study investigates the regenerative, antioxidant, and autophagy-regulating properties of extracellular vesicles to mitigate cisplatin-induced testicular damage.

Methods: Cisplatin toxicity was induced with a 7.5mg/kg dose on day one. On day six, a single dose of extracellular vesicles ($80x10^6$/100 µl) was administered. Tissue samples were collected on day eight for analysis. Testicular morphology was examined using hematoxylin and eosin staining. Sperm motility and count were assessed. Immunohistochemistry was used to evaluate autophagy markers, SF-1, and StAR expressions. Oxidative stress markers, inhibin, and testosterone levels were measured.

Results: Cisplatin-induced damage included a reduction in germinal epithelium, interstitial edema, and degeneration in seminiferous tubules, along with a decline in sperm motility and quality. An increase in the expression of autophagy markers, including Beclin-1, p62, LC3-2, SF-1, and StAR, was observed, along with elevated MDA activity and a reduction in GSH-PX, SOD, and CAT activities. Testosterone and inhibin levels also declined. In the extracellular vesicles-treated group, tissue morphology was restored, autophagy marker expression was reduced, and SF-1 and StAR expressions were increased. Oxidative stress markers decreased, and hormone levels improved.

Conclusion: Extracellular vesicles show promise in reducing cisplatin-induced reproductive toxicity and testicular damage by modulating autophagy and oxidative stress, suggesting a potential therapeutic approach for preserving fertility in patients undergoing cisplatin chemotherapy.

Keywords: extracellular vesicles; mesenchymal stem cells; cisplatin; testicular damage; autophagy

INTRODUCTION

Cisplatin (Cis) is a potent antineoplastic drug used to treat a range of cancers. Antineoplastic agents induce oxidative stress in biological systems [1-3]. It has been reported that tissue damage in the reproductive system results from reactive oxygen species produced during Cis metabolism. Its genotoxic effects include the formation of primordial follicles and the degradation of granulosa cells in the ovaries, as well as a reduction in spermatogenesis in the testicles [3–10]. Cisplatin causes cell cycle arrest and apoptosis by creating intra- and inter-strand cross-links in the DNA double helix, which inhibits DNA synthesis [11]. Testicular damage caused by Cisplatin affects Leydig cells, seminiferous tubules, spermatogenic cells, and Sertoli cells, leading to decreased sperm count, morphology, chromatin quality, and motility [4,12].

Extracellular vesicles (EVs) are a heterogeneous family of spherical, lipid-bilayered vesicles released from endosomal compartments or shed from the cell surface. This category includes exosomes, microvesicles (MVs), and apoptotic bodies. EVs are present in extracellular fluids and various bodily fluids, including blood, urine, hydrothorax, saliva, breast milk, sperm, and cerebrospinal fluid [13, 14]. EVs contain a variety of bioactive molecules, such as proteins, lipids, mRNAs, miRNAs, tRNA, genomic DNA, cDNA, and mtDNA. As extracellular organelles, EVs play a crucial role in cellular information flow [15]. EVs initiate cell-to-cell communication by delivering their cargo of proteins, miRNAs, and enzymes to target cells. EVs are extracellular organelles that function in both the paracrine and endocrine systems [13, 16]. EVs can be phagocytosed by other cell types, endocytosed, or directly fused with other cells [17].

Autophagy is a physiological process that serves as a quality control system by degrading pathogens, long-lived proteins, damaged macromolecules, and compromised organelles [18]. The primary objective is to provide an alternative source of intracellular structures and substrates for energy production to maintain cell survival. When the cellular environment fails to supply adequate nutrients, cells must consume their internal resources to survive. Additionally, cellular stress induced by pathogen infection, hypoxia, nutritional deprivation, or reactive oxygen species (ROS) can trigger autophagy. Much research has focused on how starvation-induced autophagy supports cell survival. The independent relationship between autophagy and other cell death pathways or processes, such as apoptosis, is significant [19]. Cellular autophagy can be induced by anti-tumor drugs that eliminate tumor cells. Although several oncoproteins suppress autophagy, many tumor suppressor genes enhance it [20].

Testicular damage, oxidative stress, and the activation of apoptotic signaling pathways are primary symptoms of cisplatin (Cis)-induced gonadotoxicity [3,4,11,12]. Cisplatin's gonadotoxic effect induces apoptosis in

spermatogenic cells, including Sertoli and Leydig cells within testicular tissue. Bone marrow-derived mesenchymal stem cell (MSC) extracellular vesicles (EVs) are active paracrine components with significant potential for tissue repair, suppression of inflammatory responses, and immune system modulation [21,22]. Due to their diverse components, EVs show promise in regenerative medicine [22-24]. Although studies exist on the impact of various stem cells on testicular injury in experimental Cis studies in rats, no studies have examined the therapeutic effects of EVs. This study aimed to investigate the potential therapeutic effects of bone marrow-derived MSC EVs on Cis-induced testicular damage through the mechanisms of autophagy and oxidative stress.

METHODS

Animals and experimental groups

The study was started with eight-week-old male Wistar albino rats (weight: 150–210 g). The rats were kept in a 25°C room with a 12-hour light/dark cycle, free access to water, and a standard diet from the Experimental and Clinic Research Center at Erciyes University in Kayseri, Turkey. The adult rats were randomly divided into 4 groups, with 8 rats in each group.

Control group: untreated control.

Extracellular vesicles (EVs) group: EVs ($80x10^6$/100µl) [25]. were injected intravenous (i.v.) in a single dose into rats on the sixth day of the experiment

Cis group: Cis (7,5 mg/kg) (Kocak Pharma Company, Turkey) was injected intraperitoneally (i.p.) in a single dose into rats on the first day of the experiment to induce Cis testicular toxicity (25 mg/50 ml) vials [26,27].

Cis+ extracellular vesicles (Cis+EVs) group: Cis (7,5 mg/kg) was injected i.p. in a single dose to rats on the first day to induce Cis. EVs (80 x 10^6/100 µl) were injected i.v. in a single dose into rats on the sixth day.

On the 8th day of the study, rats in the groups were sacrificed by cervical dislocation after testicular and blood samples were taken under xylazine (10 mg/kg, IP) and ketamine (60 mg/kg, IP) general anesthesia.

Experiments were carried out following the Declaration of Helsinki and the National Institutes of Health's (USA) Guide for the Care and Use of Laboratory Animals. All experimental protocols were approved by the Local Ethic Committee of Animal Experiments of Erciyes University (approval no. 22/185).

Isolation of EVs from Mesenchymal Stem Cells

Rat bone marrow derived mesenchymal stem cells were obtained from Erciyes University Genome and Stem Cell Institute (GENKÖK) in Turkiye. MSCs were thawed in a 37°C water bath and seeded in 75 cm² cell culture dishes.~~.~~ When the density of MSCs reached above 90%, serum-free medium was added to each cell group and secretomes of the cells were collected at the end of 24 hours.

EV~~s~~ isolation was performed from the obtained secretome using a standard commercial microvesicle quantitation kit ExoQuick-TC (#EXOTC50A-1, System Bio Sciences, USA) which is patented technology ~~(Antes T, et al)~~ and already in the market [28]. The collected secretomes were centrifuged at 3000 x g for 15 minutes, as per the kit instructions, ~~and the cells and their wastes sank to the bottom.~~ One-fifth of the volume of the supernatant was added to a fresh tube containing the supernatant and the exosome isolation solution. Vortex was used to assist homogenize it. After that, it was incubated for 24 hours at +4ºC. Following incubation, the mixture was centrifuged for 30 minutes at +4 ºC at 1500 x g. After centrifugation, the pellet was resuspended in PBS, and the supernatant was discarded. The mean size of the secretome ~~measured with~~, using a Nanoparticle Tracking Analysis system (NTA, Malvern Instrument Nanosight NS300, UK). According to the manufacturer's software manual (NanoSight NS300 User Manual, MAN0541-01-TR-00, 2017) adjustments were made and the measurement was completed. As a result of the analysis using the nanoparticle device, secretomes with an average diameter of 81.6 nm were found (Fig. 1). The Glomax Multi ELISA absorbance reader (Promega, USA) was used to read absorbance values at 450 nm following the quantification process that followed the kit's instructions. The amount of EVs was calculated using standards [29]. EVs diluted at 80 x 10^6/100 μl were injected into the EVs and Cis+EVs groups of rats in the study.

Figure 1. Nanoparticle Tracking Analysis

Assessment of sperm parameters

The epididymis was triturated with scissors in 5 mL of 0.9% saline and processed using a rocker for ten minutes, followed by a two-minute incubation at room temperature. For analysis, 10 µL of the supernatant containing epididymal sperm was diluted with paraformaldehyde and sodium citrate solution to a total volume of 990 µL. Subsequently, approximately 10 µL of this diluted mixture was transferred to a Neubauer chamber (hemocytometer) and examined under light microscopy at 400x magnification. The pelleted cells on the chamber's

surface were then counted. Sperm concentration was determined using the counted cells and the hemocytometer's dimensions, expressed in millions of sperm per mL, following Badkoobeh et al. (2013).

For sperm morphology assessment, around 20 µL of sperm suspension was evenly spread onto microscope slides, which were then air-dried and stained with eosin-nigrosin (1% eosin Y and 5% nigrosine). After staining and drying, the slides were examined using an optical microscope (CX31, Olympus Optical of Brazil Ltda, São Paulo, Brazil) at 400x magnification. A differential count of 200 spermatozoa per slide was conducted, noting changes in the head, middle piece, and tail. Results were expressed as the percentage of normal cells, following Badkoobeh et al. (2013) [30].

Histopathological analysis

The testicles were fixed in 10% neutral formalin for 48 hours, treated with ethanol, and then embedded in paraffin blocks for histological examinations. Sections 5 µm thick were then cut from a paraffin block (Leica, RM 2000, Germany) and stained with hematoxylin and eosin (H&E) to examine the general histomorphological structure of testis tissuesStandard light microscopy was employed to analyze and assess the testicular tissue under blinded conditions. The testicular tissue was scored according to Johnsen's criteria after being stained with H&E. [31] The Johnsen’s testicular biopsy score (JBTS) was determined by sampling five locations in each section's 20 seminiferous tubules. According to the number of cells and how mature they were, JBTS was calculated for each tubule (*Table 1*). Moreover, as a measure of damage, the diameter of the seminiferous tubule was measured at a scale of 200 x using the ImageJ software (Bethesda, MD). For the rat, the typical tubule diameter was estimated after measuring the seminiferous tubule diameters in 10 distinct places.

Table 1 Johnsen testicular biopsy score

Immunohistochemistry

The streptavidin-biotin-peroxidase method was used for the immunohistochemistry staining kit (Lab VisionTM UltraVisionTM Large Volume Detection System: anti-polyvalent, HRP, TA-125-HL). Beclin-1, p62, LC3-2, steroidogenic factor-1 (SF-1), and steroidogenic acute regulatory protein (StAR) expressions in testicular tissues were identified using this technique. Testes were cut into 5-µm sections, deparaffinized in xylene, and then rehydrated using a graduated ethanol series. After rehydration, the sections were washed in deionized water,

phosphate-buffered saline (PBS) was used to rinse the sections, and then 3% hydrogen peroxide was applied for 5 minutes. All sections were then saturated in 5% sodium citrate buffer and heated at for 5 minutes in a microwave twice at 95^{0}C to initiate antigen retrieval. Beclin-1 primary antibody diluted 1:500 (Novus, NB500-249), p62 primary antibody diluted 1:200 (Anti-SQSTM-1, H00008878), LC3-2 primary antibody diluted 1:750 (Cell Signaling, 12741 S), SF-1 primary antibody diluted 1:150 (NBP2-46247; Novus Biologicals), and StAR primary antibody diluted 1:250 (bs-3570R; Bioss Inc.) were all applied to the sections in an immunohistochemical chamber for an overnight duration at 4°C. Following that, the sections were incubated with biotinylated secondary antibodies for 10 minutes in a humid environment. Then, after the washing process, they were treated with 3,3'-diaminobenzidine tetrahydrochloride substrate (TA-060-HDX, Thermo Fisher Scientific, Waltham, MA, USA) to make the immunoreactivity visible. Gill's hematoxylin was used as a counterstain on the sections to enhance nuclear staining. Under a light microscope, each section was evaluated, and microscopic images of five different testicular tissue regions were obtained. Beclin-1, LC3-2, p62, SF-1, and StAR immunoreactivity intensities were measured using the Image J software (NIH; Washington, USA).

Elisa

Testicular tissues stored at -80 ºC for biochemical evaluation were transferred to PBS (0.01 M, pH 7.4) for homogenate preparation. Each homogenized sample was centrifuged at 4 ºC at 1500 x g for 10 min. For the ELISA method, the obtained supernatants were aliquoted. Glutathione Peroxidase (GSH-PX) (Sunred Bio, Cat. No. 201-11-5104), Superoxide Dismutase (SOD), Catalase (CAT) (Sunred Bio, Cat. No. 201-11-5106), and tumor necrosis factor-alpha (TNF-α) (Sunred Bio, Cat. No. 201-11-0765) were measured to determine the antioxidant levels of testicular tissue. The levels of serum inhibine (INHB) and testosterone were assessed. Furthermore, the level of malondialdehyde (MDA) (Sunred Bio, Cat. No. 201-11-0157) was measured in the testis tissue.

Statistical Analysis of the Data

The GraphPad Prism 9 program was used to evaluate the outcomes. The Shapiro-Wilk test was performed to determine if the data were normally distributed. A one-way analysis of variance (ANOVA) was used to analyze data that was regularly distributed. The source of the difference was then confirmed using the Tukey posthoc test. The significance level was set at $P < 0.05$.

RESULTS

EVs reduced the pathological changes in Cis-damaged testicular tissues

EVs reduced pathological changes in cisplatin (Cis)-damaged testicular tissues. Evaluation of testicular sections stained with H&E revealed that the control group's seminiferous tubules were organized into various stages of spermatogenesis and germinal epithelium, comprising spermatogonia, spermatids, and spermatocytes, with Leydig cells observed in the interstitial space. The Cis group showed degenerative alterations in the seminiferous tubules, including dilatation, reduced germ cell numbers, irregularities, and lumen shedding. In the EVs groups, the seminiferous tubules were regularly arranged, similar to the control group. Although edema was present in the Cis+EVs group, tubule degeneration was reduced, and the germinal epithelium appeared more regular. (*Fig. 2*).

Fig. 2 Histology of testis sections

The experimental group's testicular injuries were scored using JTBS (*Table 1*). The Cis group scored significantly lower than the other groups. There is a significant difference between Cis and Cis+EVs ($p<0.001$ for all comparisons) (*Fig. 3a*).

Seminiferous tubule diameters were also evaluated to assess testicular injury. The Cis group had significantly smaller seminiferous tubule diameters than the control group and EVs group. Seminiferous tubule diameter increased statistically in the Cis+EVs group compared to the Cis group. ($p<0.05$). There is a significant difference between the control, EVs, Cis ($p<0.001$), and Cis+EVs groups ($p<0.05$). (*Fig. 3b*)

Fig. 3 JTBS and Tubule Diameters

The Effect of EVs Sperm Quality Analysis

Following Cisplatin (Cis) therapy, there was a significant decrease in sperm count compared to the control group ($p = 0.000$) and the EVs group ($p = 0.007$). A significant increase was observed between the Cis and Cis+EVs groups ($p < 0.001$) (*Table 2*). Sperm motility analysis showed a significant decrease post-Cis therapy when compared to both the control and EVs groups ($p < 0.05$). Although the Cis+EVs group exhibited an increase in motility compared to the Cis group, the difference was not statistically significant. (*Table 2*).

Table 2 Sperm count and motility

Cis-induced autophagy is supressed by EVs

This research investigated the contribution of MSC-derived EVs on the expression levels of autophagy proteins Beclin-1, p62, and LC3-2 following Cisplatin (Cis) administration. In the control group, immunohistochemical evaluation of autophagy markers showed weakly positive cytoplasmic reactivity (*Fig. 4).* The Cis group exhibited a substantial increase in immunoreactivity intensity for Beclin-1, p62, and LC3-2 compared to the control group ($p < 0.001$ for all comparisons). In EVs-treated rats, there was a significant decrease in the autophagy markers Beclin-1 ($p < 0.05$), p62, and LC3-2 ($p < 0.001$) following Cis treatment (*Fig. 5*).

Fig. 4 Immunohistochemical analysis of testis

Fig. 5 İmmunoreactivity intensity of autophagy proteins

SF-1 and StAR İmmunoreactivity

SF-1 has been shown to modulate the expression of the key protein StAR, which is crucial for testosterone synthesis. In the interstitial area, Leydig cells in the control group expressed SF-1 and StAR immunoreactivity (*Fig. 6a*). The Cis group exhibited significantly lower immunoreactive intensity in Leydig cells compared to the control group ($p < 0.001$). In the Cis+EVs group, rats treated with EVs after Cis administration showed a significant increase in SF-1 immunoreactivity in Leydig cells ($p < 0.05$). However, StAR expression in the Cis+EVs group did not show a significant increase (*Fig. 6b*).

Fig. 6 Immunohistochemical analysis of SF-1 and StAR expression in interstitial area of testes a,b

Effect of EVs on testicular oxidative stress and serum hormone levels

Testicular oxidative stress injury brought on by Cis was discovered by measuring antioxidant and oxidant enzyme levels in this study. When compared to the control group, testicular GSH-PX concentration decreased significantly ($p<0.05$), and oxidative stress was dramatically initiated, as indicated by an elevated MDA level ($p<0.01$). However, compared to controls, it decreased activity, SOD, and CAT ($p<0.001$). MDA production was reduced, and MDA imbalance was improved in the Cis+EVs group treated with EVs following Cis ($p<0.001$). EVs-therapy increased GSH-PX concentration in the Cis+EVs group rats ($p<0.05$) and significantly decreased oxidative stress by decreasing testicular MDA ($p<0.001$). When compared to the control group, there was a significant rise in testicular TNF-α expression in the Cis-treated group ($p<0.05$). The EVs-treated Cis+EVs group blocked the increase in testicular TNF-α expression compared to the Cis group, but there was no statistically significant difference (*Fig. 7*).

Fig. 7 Statistical analysis of testicular oxidative stress

Reduced SF-1 and StAR immunoreactivity in Leydig cells resulted in decreased testosterone levels. ~~INHB regulates sperm production by inhibiting the pituitary gland from secreting too much follicle-stimulating hormone (FSH). The presence of androgens in the blood increases inhibin levels, promoting spermatogenesis. Reduced testosterone levels also have a deleterious impact on INHB levels.~~ Cis therapy decreased serum testosterone ($p<0.005$) and INHB levels ($p<0.001$) significantly ($p<0.001$). When compared to the Cis group, EVs therapy increased serum testosterone ($p<0.005$) and INHB levels ($p<0.001$) (*Fig. 8*).

Fig. 8 Statistical analysis of serum hormone levels

DISCUSSION

Cisplatin (Cis), a chemotherapy drug used to treat various cancers, causes damage to testicular tissue despite its potent antitumor activity. Because chemotherapy agents target dividing cells, the spermatogenesis process is particularly affected. Previous studies have reported that exposure to Cis reduces reproductive function

[2,9,10,12,32]. This study's histological, immunohistochemical, and biochemical results demonstrated the damaging impact of Cis on testicular tissue.

Antitumor drugs that destroy cancer cells, such as Cis, damage tissue by inducing autophagy and apoptosis in cells [1-3,5,8-12,26,27]. Extracellular vesicles (EVs) derived from bone marrow mesenchymal stem cells (MSCs) have shown therapeutic potential in various studies on tissue damage and degenerative disorders [13,17,33–35]. EVs function as extracellular organelles with paracrine/endocrine roles, transporting proteins, miRNAs, and enzymes to target cells [17]. Many studies have reported that EVs have anti-apoptotic, anti-inflammatory, and regenerative effects [15,33–35].

Cis therapy altered testicular morphology, induced autophagy, and decreased hormone levels, sperm count, and oxidative stress. A single dose of EVs therapy demonstrated significant regenerative and antioxidant effects, as well as increased autophagic activity. Although hormone levels increased significantly, sperm motility and count remained unaffected. The Cis group exhibited degeneration, atrophied tubular epithelium, cavities, and dilation of seminiferous tubules. A reduction in tubule spermatogenic cells, germ cell infiltration into the lumen, and interstitial edema were observed. These findings were corroborated by a decrease in the JTBS score and tubule diameter. Previous studies reported that seminiferous tubule diameters, germinal epithelial thickness, and testosterone synthesis decreased in Cis-treated rats, potentially leading to testicular atrophy and infertility [36–38].

EVs are highly effective active paracrine components that play a vital role in cell communication and have a high potential for tissue repair [21]. EVs establish cell-cell interactions by delivering proteins, miRNAs, and enzymes to target cells, functioning as extracellular organelles with paracrine/endocrine roles [16]. EVs proteins and microRNAs have a wide range of biochemical and physiological functions, including communication, structure and interactions, inflammation, tissue repair and regeneration, and metabolism [14,15,34]. EVs treatment reduced seminiferous tubule degeneration and restored germinal epithelial cell structure. However, edema persisted in this group. Additionally, the JTBS score and tubule diameters increased in this group.

Cisplatin (Cis), an alkylating agent, cross-links the inner and outer DNA chains, generating adduct forms that manifest as twisted DNA strands. This mechanism presumably affects sperm and testicular structure [36]. Studies have shown that Cis treatment significantly decreases sperm count and motility in rat testes [26,37,38]. Cis damages Sertoli cells and weakens the blood-testis barrier, leading to DNA damage in germ cells at various developmental stages. This situation adversely affects the functional capacity of sperm stored and matured in the epididymis. Elevated ROS levels and increased apoptosis result in reduced sperm quantity and quality. These

effects of Cis become more pronounced with higher doses and repeated applications [7, 26, 39, 40]. In this study, Cis therapy significantly decreased sperm motility and count. A significant increase in sperm count was observed in the Cis+EVs group that received EVs therapy, although sperm motility did not significantly improve. Sperm count and motility increased when EVs were administered as a single dose on the sixth day following Cis therapy, but motility did not significantly improve.

Because EVs, especially exosome uptake, are dependent on intracellular and microenvironmental acidity, and tissue injury is often characterized by tissue acidosis, exosomes are preferentially endocytosed by cells in injured tissue. This implies that exosomes could home to injured tissues [41]. Furthermore, EVs are small, nanometer-sized particles that can easily be transported via blood and other bodily fluids, as indicated by the quantity of EVs found in most biological fluids. EVs can interact with cells in both paracrine and endocrine manners [13, 14]. As a result, EVs have the biophysical capacity to orchestrate MSC interactions with a variety of cell types in both local and distant environments. However, the ability to evoke appropriate cellular responses after tissue injury to restore and maintain tissue microenvironment homeostasis would depend on the biochemical capability of their protein and RNA cargo [13, 14, 33].

Autophagy, which is essential for cellular adaptability and survival, can be triggered by various stressors, including metabolic stress, endoplasmic reticulum (ER) stress, and chemotherapeutic drugs [42]. Spermatogenesis requires a significant amount of energy. During this process, factors such as starvation, chemical exposure, and radiation can have adverse effects. Studies have shown the connection between apoptotic and autophagic molecular mechanisms under these pathophysiological conditions [43, 44]. Cisplatin-induced oxidative stress and tissue acidosis in testicular tissue stimulate both the apoptotic pathway and autophagy. Autophagy induced by Cisplatin in the testis is evidenced by increased levels of Beclin-1, LC3-2, and p62 proteins. A single dose of EVs therapy significantly improved tissue repair by regulating Cisplatin-induced autophagy. Previous studies have shown that adipose-derived MSC-EVs provide neuroprotection and enhance neurological recovery by inhibiting ischemia-induced autophagy [45]. Adipose-derived MSC exosomes protect against testicular ischemia/reperfusion injury in rats through antioxidant, anti-inflammatory, and anti-apoptotic mechanisms [39]. It has also been reported that antitumoral drugs trigger the autophagy process [20]. EVs have demonstrated immunoregulatory, anti-inflammatory, regenerative, and anti-apoptotic capacities in therapeutic applications; however, whether these characteristics regulate autophagy remains unclear [17].

Oxidative stress, defined as an imbalance between free radicals and the antioxidant defense system, plays a crucial role in disease development [46]. Chemotherapy drugs cause mitochondrial damage, leading to increased reactive oxygen species (ROS) and oxidative stress [47]. This ROS accumulation acts as a chemoattractant, attracting MSCs and EVs to the damage site [48]. Antioxidant enzyme levels such as GSH-PX, MDA, SOD, and CAT were evaluated in male rats to assess the therapeutic effects of EVs on testicular toxicity caused by Cisplatin-induced oxidative stress. The Cisplatin group showed significantly lower GSH-PX, SOD, and CAT activities in testicular homogenate compared to the control group. Cisplatin increased MDA levels, a marker of lipid peroxidation, and significantly raised TNF-α, an inflammatory marker [2,3,10,26,27,36]. The EVs-treated Cisplatin group showed significant improvements in GSH-PX, SOD, and CAT activities and a significant reduction in MDA levels. Although single-dose EVs reduced TNF-α levels, the decrease was not significant. Elevated TNF-α levels impair spermatogenesis and reduce testosterone levels, with increased ROS production contributing to this process through apoptosis [50,51,52]. Studies have demonstrated that EVs and exosomes can penetrate bodily barriers to reach damaged areas [34, 53, 54]. EVs with anti-apoptotic, anticancer, and antioxidant properties have been shown to reduce oxidative stress at the damage site [17,44,54,55].

The primary functions of the testicles are the production of testosterone and sperm [56]. Leydig cells in the interstitial tissue of the testes secrete testosterone [57]. In this study, the Cisplatin group had lower testosterone levels, correlating with decreased SF-1 and StAR protein expression. INHB is a key hormone regulating reproductive function and FSH~~, released by Sertoli cells in the testes~~ [58,59]. FSH stimulates seminiferous tubules and Sertoli cells, initiating spermatogenesis, which requires testosterone [60].

INHB and FSH secretion are negatively correlated during Sertoli cell development and spermatogenesis. Decreased serum INHB indicates sperm damage and infertility, thus serving as a measure of male spermatogenesis [61,62]. Inhibins also act as growth factors in Leydig cells via paracrine mechanisms, indirectly regulating spermatogenesis along with testosterone [63]. Previous research has shown that Cisplatin intake directly affects Leydig cells, causing cellular death and reduced secretory activity [27,64]. Due to damaged seminiferous tubule epithelium and low testosterone levels, serum INHB levels were significantly lower in the Cisplatin group compared to the control group. INHB levels significantly increased in the EVs-treated group after Cisplatin, supported by tissue regeneration in the seminiferous tubule and interstitial areas.

CONCLUSION

EVs offer numerous advantages as a cell-free therapy and have shown potential in treating various human diseases in recent years. They are natural, can be derived from a person's own cells, and targeted to specific cells or tissues. This study investigated the therapeutic efficacy of bone marrow-derived MSC EVs on Cisplatin-induced testicular damage in rats. EVs, possessing regenerative and anti-cancer properties, were found to mitigate Cisplatin-induced testicular damage, suggesting their potential as a treatment for such damage and germ cell layer degeneration. This study indicates that a single dose of EVs can ameliorate Cisplatin-induced testicular damage by reducing oxidative stress and regulating autophagy. Further studies should explore repeated doses, different dosing regimens, and more localized applications for more effective and long-term results. Additionally, research focusing on the molecular regulation of autophagy may provide a deeper understanding of its therapeutic effects.

ACKNOWLEDGMENT

We thank the staff of the Erciyes University ERU Experimental Research and Application Centre for their care of the rats used in this study. We also appreciate the support from the Gevher Nesibe Genome and Stem Cell Institute (GENKÖK) of Erciyes University in generating BM-MSCs and the EVs derived from these cells. This study received no funding.

CONFLİCT OF INTEREST STATEMENT

The authors declare no relevant competing interests.

ETHİCS STATEMENT

The experiments were conducted in accordance with The ARRIVE guidelines (Animal Research: Reporting of In Vivo Experiments). All experimental protocols were approved by the Local Ethic Committee of Animal Experiments of Erciyes University (approval no. 22/185)in Turkiye.

DATA AVAİLABİLİTY

The data in this study were obtained from experiments, not from the internet, and therefore are not suitable for data sharing.

AUTHORS' CONTRIBUTIONS

H.T.Y.: conceptualization, data curation, resources, writing-original draft, and writing-review and editing.

K.T.K.: data curation, formal analysis, methodology, and software.

N.B.: methodology, supervision, and validation.

Z.B.G.: conceptualization, methodology, and validation.

O.C.M.: formal analysis, methodology, supervision, and validation.

E.K.: formal analysis, methodology, and software.

G.O.O.: Investigation, project administration, and validation.

A.Y.: conceptualization, supervision, visualization, writing, and review and editing.

FUNDING

The authors did not receive support from any organization for the submitted work.

REFERENCES

1- McSweeney KR, Gadanec LK, Qaradakhi T, Ali BA, Zulli A, Apostolopoulos V. Mechanisms of cisplatin-induced acute kidney injury: Pathological mechanisms, pharmacological ınterventions, and genetic mitigations. Cancers. Basel. 2021 Mar 29;13(7):1572. doi: 10.3390/cancers13071572. PMID: 33805488; PMCID: PMC8036620

2- Moradi M, Goodarzi N, Faramarzi A, Cheraghi H, Hashemian AH, Jalili C. Melatonin protects rats testes against bleomycin, etoposide, and cisplatin-induced toxicity via mitigating nitro-oxidative stress and apoptosis. Biomed Pharmacother. 2021 Jun;138:111481. doi: 10.1016/j.biopha.2021.111481. Epub 2021 Mar 19. PMID: 33752059

3- Sallam AO, Rizk HA, Emam MA, Fadl SE, Abdelhiee EY, Khater H. et al. The ameliorative effects of l-carnitine against cisplatin-induced gonadal toxicity in rats. Pak Vet J. 2021;41(1):147-151. DOI: 10.29261/pakvetj/2020.082.

4- Mackie EJ, Radford M, Shalet SM. Gonadal function following chemotherapy for childhood Hodgkin's disease. Med Pediatr Oncol. 1996 Aug;27(2):74-8. doi: 10.1002/(SICI)1096-911X(199608)27:2<74:AID-MPO2>3.0.CO;2-Q. PMID: 8649323.

5- Dasari S, Njiki S, Mbemi A, Yedjou CG, Tchounwou PB. pharmacological effects of cisplatin combination with natural products in cancer chemotherapy. Int J Mol Sci. 2022 Jan 28;23(3):1532.

6- Li X, Kang X, Deng Q, Cai J, Wang Z. Combination of a GnRH agonist with an antagonist prevent sflare-upeffects and protects primordial ovarian follicles in therat ovary from cisplatin-induced toxicity: a controlled experimental animal study. Reprod Biol Endocrino. 2013;11-16.

7- Boekelheide K. Mechanisms of toxic damage to spermatogenesis. J Natl Cancer Inst Monogr. 2005;(34):6-8. doi: 10.1093/jncimonographs/lgi006. PMID: 15784812.

8- Amin A, Hamza A. Effects of ginger and roselle on cisplatin induced reproductive toxicity in rats. Asian J Androl. 2006;8(5):607–12.

9- Amin A, Hamza AA, Kambal A, Daoud S. Herbal extracts counteract cisplatin-mediated cell death in rat testis. Asian J Androl. 2008 Mar;10(2):291-7. doi: 10.1111/j.1745-7262.2008.00379.x. Epub 2007 Dec 20. PMID: 18097501.

10- Mesbahzadeh B, Hassanzadeh-Taheri M, Aliparast Ms, Baniasadi P, Hosseini M. The protective effect of crocin on cisplatin-induced testicular impairment in rats. BMC Urol. 2021;21:117. Available from: https://doi.org/10.1186/s12894-021-00889-2.

11- Sueishi K, Mishima K, Makino K, Itoh Y, Tsuruya K, Hirakata H. Protection by a radical scavenger edaravone against cisplatin-induced nephrotoxicity in rats. Eur J Pharmacol. 2002;451:203–8.

12- Whirledge SD, Garcia JM, Smith RG, Lamb DJ. Ghrelin partially protects against cisplatin-induced male murine gonadal toxicity in a GHSR-1a-dependent manner. Biol Reprod. 2015 Mar;92(3):76.

13- Chen B, Li Q, Zhao B, Wang Y. Stem Cell-Derived Extracellular Vesicles as a Novel Potential Therapeutic Tool for Tissue Repair. Stem Cells Transl Med. 2017 Sep;6(9):1753-1758. doi: 10.1002/sctm.16-0477. Epub 2017 Jun 27. PMID: 28653443; PMCID: PMC5689748.

14- Maia J, Caja S, Strano Moraes MC, Couto N, Costa-Silva B. Exosome-based cell-cell communication in the tumor microenvironment. Front Cell Dev Biol. 2018 Feb;6:18.

15- Háněľová, K., Raudenská, M., Masařík, M. et al. Protein cargo in extracellular vesicles as the key mediator in the progression of cancer. Cell Commun Signal 22, 25 (2024). https://doi.org/10.1186/s12964-023-01408-6.

16- Gougheri, K. A., Ahmadi, A., Ahmadabadi, M. G., Babajani, A., Yazdanpanah, G., Bahrami, S., ... & Niknejad, H. (2023). Exosomal Cargo: Pro-angiogeneic, anti-inflammatory, and regenerative effects in ischemic and non-ischemic heart diseases-A comprehensive review. Biomedicine & pharmacotherapy= Biomedecine & pharmacotherapie, 168, 115801.

17- Rani S, Ryan AE, Griffin MD, Ritter T. Mesenchymal stem cell derived extracellular vesicles: toward cell-free therapeutic applications. Molecular Therapy. 2015;23:812-823.

18- Anding AL, Baehrecke EH. Autophagy in Cell Life and Cell Death. Curr Top Dev Biol. 2015;114:67-91. doi: 10.1016/bs.ctdb.2015.07.012. Epub 2015 Sep 9. PMID: 26431564.

19- Galluzzi L, Vitale I, Abrams JM, Alnemri ES, Baehrecke EH, Blagosklonny MV. Molecular definitions of cell death subroutines: Recommendations of the nomenclature committee on cell death. Cell Death and Differentiation. 2012;19(1):107–120.

20- Xiao G. Autophagy and Nf-Kb: Fight for fate. Cytokine Growth Factor Rev. 2007 Jun-Aug;18(3-4):233-43.

21- Heydari Z, Peshkova M, Gonen ZB, Coretchi I, Eken A, Yay AH, Dogan ME, Gokce N, Akalin H, Kosheleva N, Galea-Abdusa D, Ulinici M, Vorojbit V, Shpichka A, Groppa S, Vosough M, Todiras M, Butnaru D, Ozkul Y, Timashev P. EVs vs. EVs: MSCs and Tregs as a source of invisible possibilities. J Mol Med (Berl). 2023 Feb;101(1-2):51-63. doi: 10.1007/s00109-022-02276-2. Epub 2022 Dec 17. PMID: 36527475; PMCID: PMC9759062.

22- Toh WS, Zhang BIN, Lai RC, Lim SK. Immune regulatory targets of mesenchymal stromal cell exosomes/small extracellular vesicles in tissue regeneration. Cytotherapy. 2018;20:(12):1419-1426.

23- Reiner, A. T., Witwer, K. W., Van Balkom, B. W., De Beer, J., Brodie, C., Corteling, R. L., ... & Lim, S. K. Concise review: developing best-practice models for the therapeutic use of extracellular vesicles. Stem cells translational medicine. 2017;6(8), 1730-1739.

24- Raposo G and Stoorvogel W. Extracellular vesicles: exosomes, microvesicles, and friends. The Journal of Cell Biology. 2013;200:373-383.

25- Helwa I, Cai J, Drewry MD, Zimmerman A, Dinkins MB, Khaled ML, Seremwe M, Dismuke WM, Bieberich E, Stamer WD, Hamrick MW, Liu Y. A Comparative Study of Serum Exosome Isolation Using Differential Ultracentrifugation and Three Commercial Reagents. PLoS One. 2017 Jan 23;12(1):e0170628.

26- Abdel-Latif R, Fathy M, Anwar HA, Naseem M, Dandekar T, Othman EM. Cisplatin-induced reproductive toxicity and oxidative stress: Ameliorative effect of kinetin. Antioxidants. 2022;11(5):863. Available from: https://doi.org/10.3390/antiox11050863.

27- Afsar T, Razak S, Khan MR, Almajwal A. Acacia hydaspica ethyl acetate extract protects against cisplatin-induced DNA damage, oxidative stress and testicular injuries in adult male rats. BMC Cancer. 2017;17(1):883.

28- Antes T, et al. Methods for Microvesicle Isolation and Selective Removal. Patent No.: US 9,005,888 B.

29- Bahar D, Gonen ZB, Gumusderelioglu M, Onger ME, Tokak EK, Ozturk-Kup F, et al. Repair of rat calvarial bone defect by using exosomes of umbilical cord-derived mesenchymal stromal cells embedded in chitosan/hydroxyapatite scaffolds. Int J Oral Maxillofac Implants. 2022 Sep-Oct;37(5):943-950.

30- Badkoobeh P, Parivar K, Kalantar SM, Hosseini SD, Salabat A. Effect of nano-zinc oxide on doxorubicin-induced oxidative stress and sperm disorders in adult male Wistar rats. Iran J Reprod Med. 2013 May;11(5):355-64.

31- Johnsen SG. Testicular biopsy score count–a method for registration of spermatogenesis in human testes: normal values and results in 335 hypogonadal males. Hormones. 1970;1:2–25.

32- Shabani R, Ashtari K, Behnam B, Izadyar F, Asgari H, Asghari Jafarabadi M, et al. In vitro toxicity assay of cisplatin on mouse acute lymphoblastic leukaemia and spermatogonial stem cells, Andrologia. 2016;48:(5):584–94.

33- Fujii S, Miura Y. Immunomodulatory and Regenerative Effects of MSC-Derived Extracellular Vesicles to Treat Acute GVHD. Stem Cells. 2022 Nov 29;40(11):977-990. doi: 10.1093/stmcls/sxac057. PMID: 35930478.

34- Ding JY, Chen MJ, Wu LF, Shu GF, Fang SJ, Li ZY, Chu XR, Li XK, Wang ZG, Ji JS. Mesenchymal stem cell-derived extracellular vesicles in skin wound healing: roles, opportunities and challenges. Mil Med Res. 2023 Aug 17;10(1):36. doi: 10.1186/s40779-023-00472-w. PMID: 37587531; PMCID: PMC10433599.

35- Tsiapalis D, O'Driscoll L. Mesenchymal Stem Cell Derived Extracellular Vesicles for Tissue Engineering and Regenerative Medicine Applications. Cells. 2020 Apr 16;9(4):991. doi: 10.3390/cells9040991. PMID: 32316248; PMCID: PMC7226943.

36- Narayana K, Al-Bader M, Mousa A, Khan KM. Molecular effects of chemotherapeutic drugs and their modulation by antioxidants in the testis. Eur J Pharmacol 2012;674:207-216.

37- Prihatno SA, Padeta I, Larasati AD, Sundari B, Hidayati A, Fibrianto, et al. Effects of secretome on cisplatin-induced testicular dysfunction in rats. Vet. World. 2018;11:1349.

38- Gholami Jourabi F, Yari S, Amiri P, Heidarianpour A, Hashemi H. The ameliorative effects of methylene blue on testicular damage induced by cisplatin in rats. Andrologia. 2021 Feb;53(1):e13850.

39- Liu H, Shi M, Li X, Lu W, Zhang M, Zhang T, et al. Adipose mesenchymal stromal cell-derived exosomes Prevent testicular torsion injury via activating PI3K/AKT and MAPK/ERK1/2 pathways. Oxid Med Cell Longev. 2022 Jun 16;2022:8065771.

40- Hsiao CH, Ji AT, Chang CC, Chien MH, Lee LM, and Ho JHC. "Mesenchymal stem cells restore the sperm motility from testicular torsion-detorsion injury by regulation of glucose metabolism in sperm," Stem Cell Research & Therapy, vol. 10, no. 1, p. 270, 2019.

41- Lai RC, Yeo RW, Lim SK. Mesenchymal stem cell exosomes. Semin Cell Dev Biol. 2015 Apr;40:82-8.

42- Yoshida GJ. Therapeutic strategies of drug repositioning targeting autophagy to induce cancer cell death: from pathophysiology to treatment. J. Hematol. Oncol. 2017;10:67.

43- Zhang M, Jiang M, Bi Y, Zhu H, Zhou Z, Sha J. Autophagy and apoptosis act as partners to induce germ cell death after heat stress in mice. PLoS One. 2012;7(7):e41412. doi: 10.1371/journal.pone.0041412. Epub 2012 Jul 25. PMID: 22848486; PMCID: PMC3405141.

44- Gallagher LE, Williamson LE, Chan EY. Advances in Autophagy Regulatory Mechanisms. Cells. 2016 May 13;5(2):24. doi: 10.3390/cells5020024. PMID: 27187479; PMCID: PMC4931673.

45- Kuang Y, Zheng X, Zhang L, Ai X, Venkataramani V, Kilic E, Hermann DM, Majid A, Bähr M, Doeppner TR. Adipose-derived mesenchymal stem cells reduce autophagy in stroke mice by extracellular vesicle transfer of miR-25. J Extracell Vesicles. 2020 Oct;10(1):e12024. doi: 10.1002/jev2.12024. Epub 2020 Nov 11. PMID: 33304476; PMCID: PMC7710129.

46- Afsar T, Razak S, Khan MR, Almajwal A. Acacia hydaspica ethyl acetate extract protects against cisplatin-induced DNA damage, oxidative stress and testicular injuries in adult male rats. BMC Cancer. 2017 Dec 21;17(1):883.

47- Dasari S, Tchounwou PB. Cisplatin in cancer therapy: molecular mechanisms of action. Eur J Pharmacol. 2014 Oct 5;740:364-78.

48- Cocetta V, Ragazzi E, Montopoli M. Mitochondrial Involvement in Cisplatin Resistance. Int J Mol Sci. 2019 Jul 10;20(14):3384.

49- Wan M, Li C, Zhen G, Jiao K, He W, Jia X, et al. Injury-activated transforming growth factor β controls mobilization of mesenchymal stem cells for tissue remodeling. Stem Cells. 2012 Nov;30(11):2498-2511.

50- Aly HA, Eid BG. Cisplatin induced testicular damage through mitochondria mediated apoptosis, inflammation and oxidative stress in rats: Impact of resveratrol. Endocr. J. 2020;67:969–980.

51- Wang L, He Y, Li Y, Pei C, Olatunji OJ, Tang J, et al. Protective effects of nucleosides-rich extract from cordyceps cicadae against cisplatin ınduced testicular damage. Chem Biodivers. 2020 Nov; 17(11):e2000671.

52- Ferreiro ME, Amarilla MS, Glienke L, Méndez CS, González C, Jacobo PV, et al. The inflammatory mediators TNF-α and nitric oxide arrest spermatogonia GC-1 cell cycle. Reprod. Biol. 2019;19:329–339.

53- Li CY, Liu SP, Dai XF, Lan DF, Song T, Wang, XY, Kong QH, Tan J, Zhang JD. The emerging role of exosomes in the development of testicular. Asian Journal of Andrology 25(5):p 547-555, Sep–Oct 2023. | DOI: 10.4103/aja2022126.

54- Tian, C., Gao, L., Rudebush, T.L., Yu, L., Zucker, I.H., 2022. Extracellular Vesicles Regulate Sympatho-Excitation by Nrf2 in Heart Failure. Circulation Research 131, 687–700.. https://doi.org/10.1161/circresaha.122.320916.

55- Guo XB, Zhai JW, Xia H, Yang JK, Zhou JH, Guo WB, et al. Protective effect of bone marrow mesenchymal stem cell-derived exosomes against the reproductive toxicity of cyclophosphamide is associated with the p38MAPK/ERK and AKT signaling pathways. Asian J Androl. 2021 Jul-Aug;23(4):386-391.

56- Hassanzadeh-Taheri M, Hosseini M. Comments on “The improvement efects of Gordonia bronchialis on male fertility of rats with diabetes mellitus induced by Streptozotocin". Pharm Sci. 2020;26(1):93–95. **doi:** 10.34172/PS.2019.60.

57- Hoivik EA, Lewis AE, Aumo L, Bakke M. Molecular aspects of steroidogenic factor 1 (SF-1). Mol Cell Endocrinol. 2010;315:27–39.

58- Ying SY. Inhibins, activins, and follistatins: gonadal proteins modulating the secretion of follicle-stimulating hormone. Endocr Rev 1988:267-93. Available from: https://doi.org/10.1210/edrv-9-2-267.

59- Han, Y., Jiang, T., Liu, A., & Liu, L. Role and regulatory mechanism of inhibin in animal reproductive system. Theriogenology. 2023;202:10-20.

60- Oduwole, O.O., Huhtaniemi, I.T., Misrahi, M. The Roles of Luteinizing Hormone, Follicle-Stimulating Hormone and Testosterone in Spermatogenesis and Folliculogenesis Revisited. Int. J. Mol. Sci. 2021, 22, 12735. https://doi.org/10.3390/ijms222312735.

61- Andersson AM, Juul A, Petersen JH, Müller J, Groome NP, Skakkebaek NE. Serum inhibin B in healthy pubertal and adolescent boys: relation to age, stage of puberty, and follicle-stimulating hormone, luteinizing hormone, testosterone, and estradiol levels. J Clin Endocrinol Metab. 1997 Dec;82(12):3976-81. doi:10.1210/jcem.82.12.4449. PMID: 9398699.

62- Pfaff T, Rhodes J, Bergmann M, Weinbauer GF. Inhibin B as a marker of sertoli cell damage and spermatogenic disturbance in the rat. Birth Defects Res B Dev Reprod Toxicol. 2013 Feb;98(1):91-103. doi: 10.1002/bdrb.21046. Epub 2013 Jan 24. PMID: 23349029.

63- de Kretser DM, Buzzard JJ, Okuma Y, O'Connor AE, Hayashi T, Lin SY, Morrison JR, Loveland KL, Hedger MP. The role of activin, follistatin and inhibin in testicular physiology. Mol Cell Endocrinol. 2004;225(1-2):57-64.

64- Tian M, Liu F, Liu H, Zhang Q, Li L, Hou X, et al. Grape seed procyanidins extract attenuates Cisplatin-induced oxidative stress and testosterone synthase inhibition in rat testes. Syst Biol Reprod Med. 2018;64:246–59.

Table 1 Johnsen testicular biopsy score

Score	Histological findings	Score	Histological findings
1	No cells in the tubular section	6	There were few spermatids (5/tubule)
2	There were only Sertoli cells	7	There were too many spermatids without any sign of difference
3	Germ cells were just as spermatogonium	8	Late spermatids without mature spermatozoa
4	There were few spermatocytes (5/tubule)	9	There were few spermatozoa (5/tubule)
5	There were too many spermatocytes	10	Exact spermatogenesis was present with a large number of spermatozoa

Table 2 Sperm count and Sperm motility

	**Control (mean ± SEM)**	**EVs (mean ± SEM)**	**Cis (mean ± SD)**	**Cis+EVs (mean ± SD)**
**Sperm Count (million/mL)**	47.33 ± 4.93	36.16 ± 2.38$^{\$\$\$,***}$	13.00 ± 1.75***	16.00 ± 1.09***
**Sperm Motility (%)**	51.66 ± 4.77	50.83 ± 8.20$^{\&\&}$	21.66 ± 6.91**	44.16 ± 7.35

Table 2. Sperm count and motility. Effect of exosomes on **s**perm count (million/mL), and sperm motility **(%)** in testis tissues**.** *** : Control versus Cis, Control versus Cis+EVs, Control versus EVs, Cis+EVs versus Cis (p<0.001), $^{\$\$\$}$: Cis versus EVs (p<0.001), $^{\&\&}$: Cis versus EVs (p<0.05), ** : Control versus Cis (p<0.05).

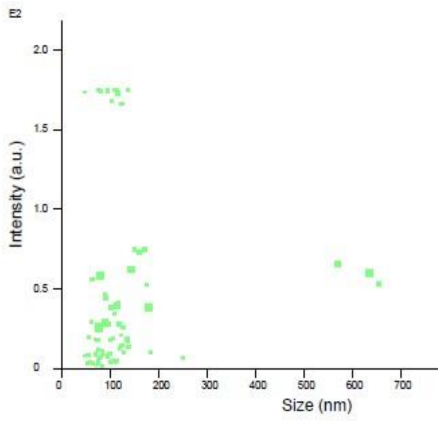

Fig. 1 Nanoparticle Tracking Analysis

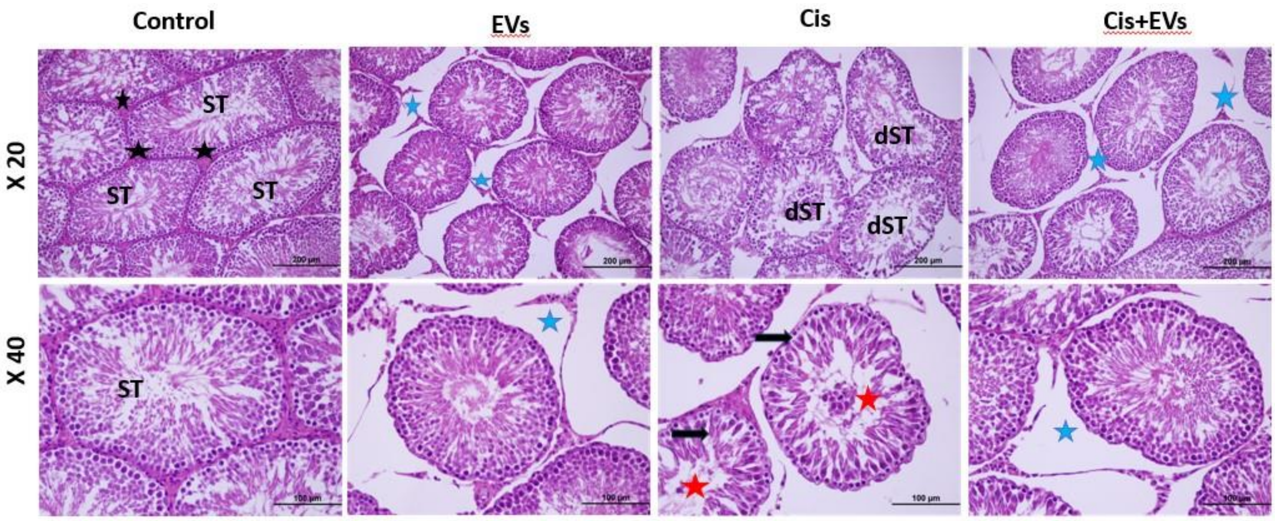

Fig. 2 Histology of testis sections (Olympus BX51, Tokyo, Japan X20: Scale bar; 200 µm, X40: Scale bar; 100 µm). Effects of EVs on seminiferous tubules (ST) and interstitial tissue with Leydig cells (black asterisks) in testis tissues. Representative Hematoxylin and Eosin stained sections from indicated groups. Normal histology was observed in the control group. The Cis group experienced degenerative seminiferous tubule (dST) features. Black arrow; decreased number of germ cells, Red asterisks; lumen shedding, Blue asterisks; edema

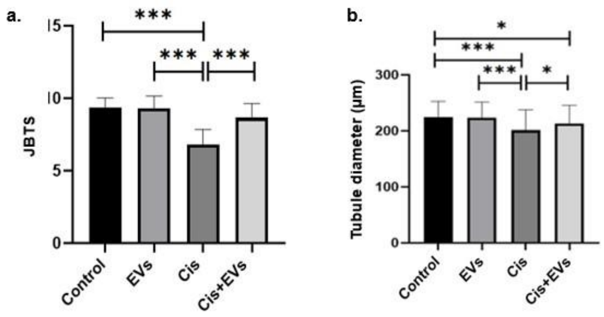

Fig. 3. JTBS and Tubule Diameters a. The general Johnsen's testicular biopsy scoring for all groups. **b.** Seminiferous tubule diameter measurement for all groups. *, p<0.05, **; p<0.01, **; p<0.001

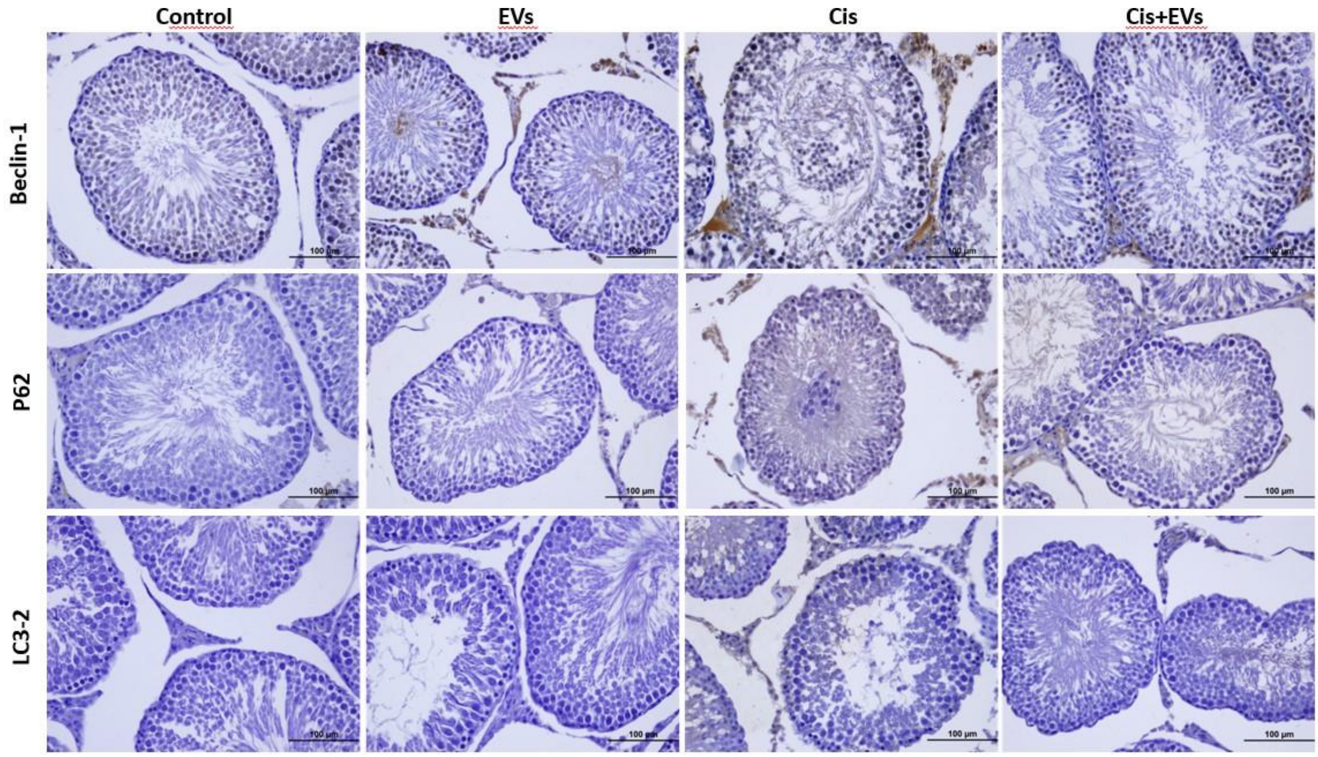

Fig. 4. Immunohistochemical analysis of testis sections (Olympus BX51, Tokyo, Japan. X40: Scale bar; 100 µm). Levels of autophagy markers in the testes of control and treated group. EVs treatment ameliorated expression of Beclin-1, P62, and LC3-2 levels in the testes of cisplatin-induced rats. Avidin-biotin peroxidase technique.

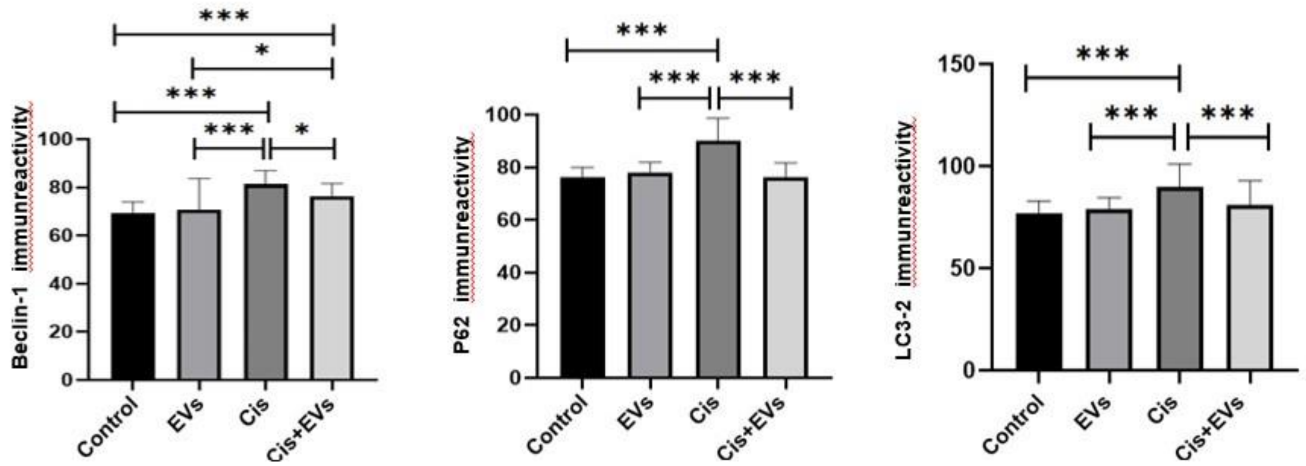

Fig. 5 İmmunoreactivity intensity of autophagy proteins Impact of EVs Beclin-1, p62, and LC3/2 in the testes tissue of rats treated with cisplatin. *,p<0.05, **p<0.01, ***p<0.001

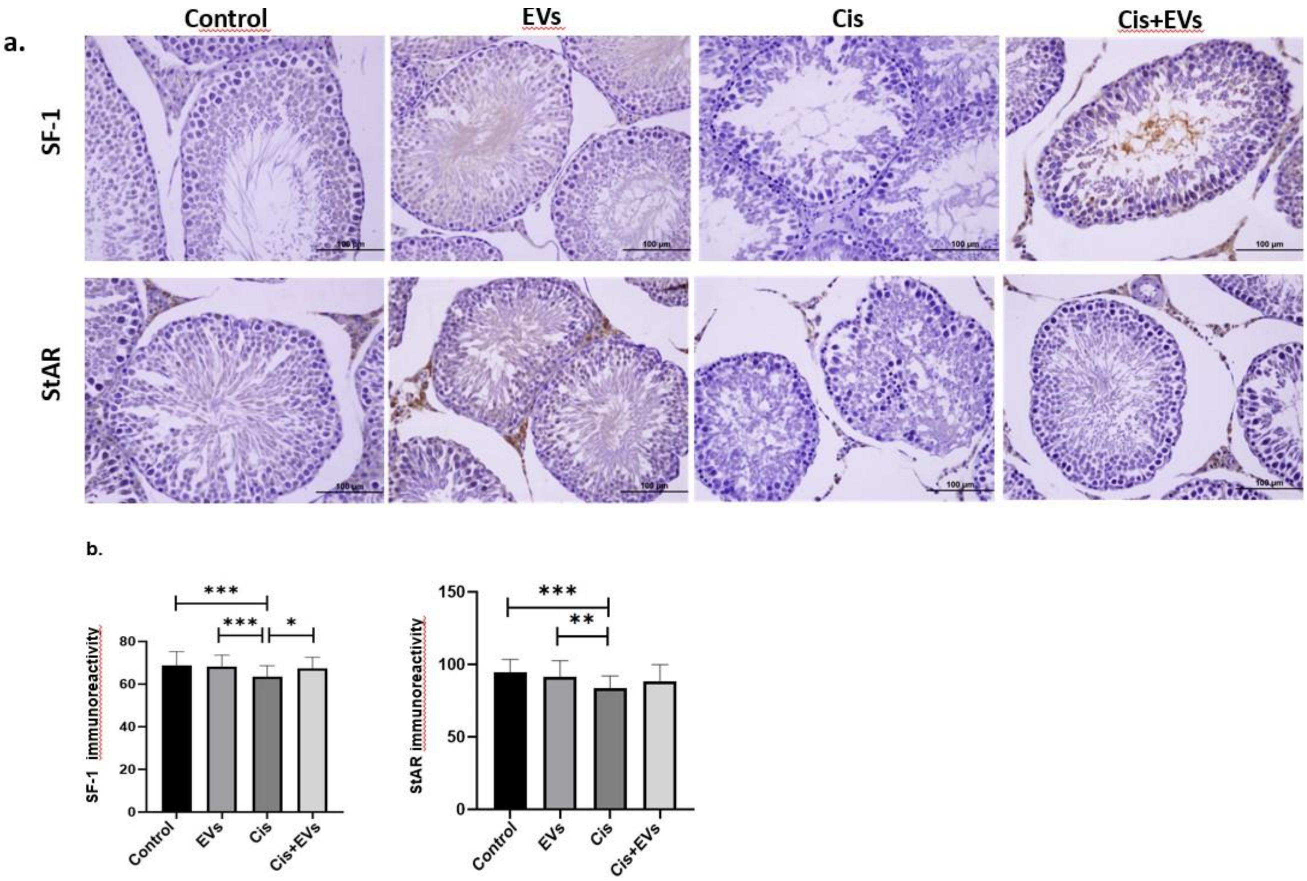

Fig. 6 Immunohistochemical analysis of SF-1 and StAR expression in interstitial area of testes (Olympus BX51, Tokyo, Japan. X40: Scale bar; 100 µm). **a.** SF-1 and StAR expression in all experimental groups is shown in the illustrative figure. Avidin-biotin peroxidase technique. **b**. Immunoreactivity intensity of SF-1 and StAR in interstitial area of testes tissue. *, p<0.05, **; p<0.01, ***; p<0.001

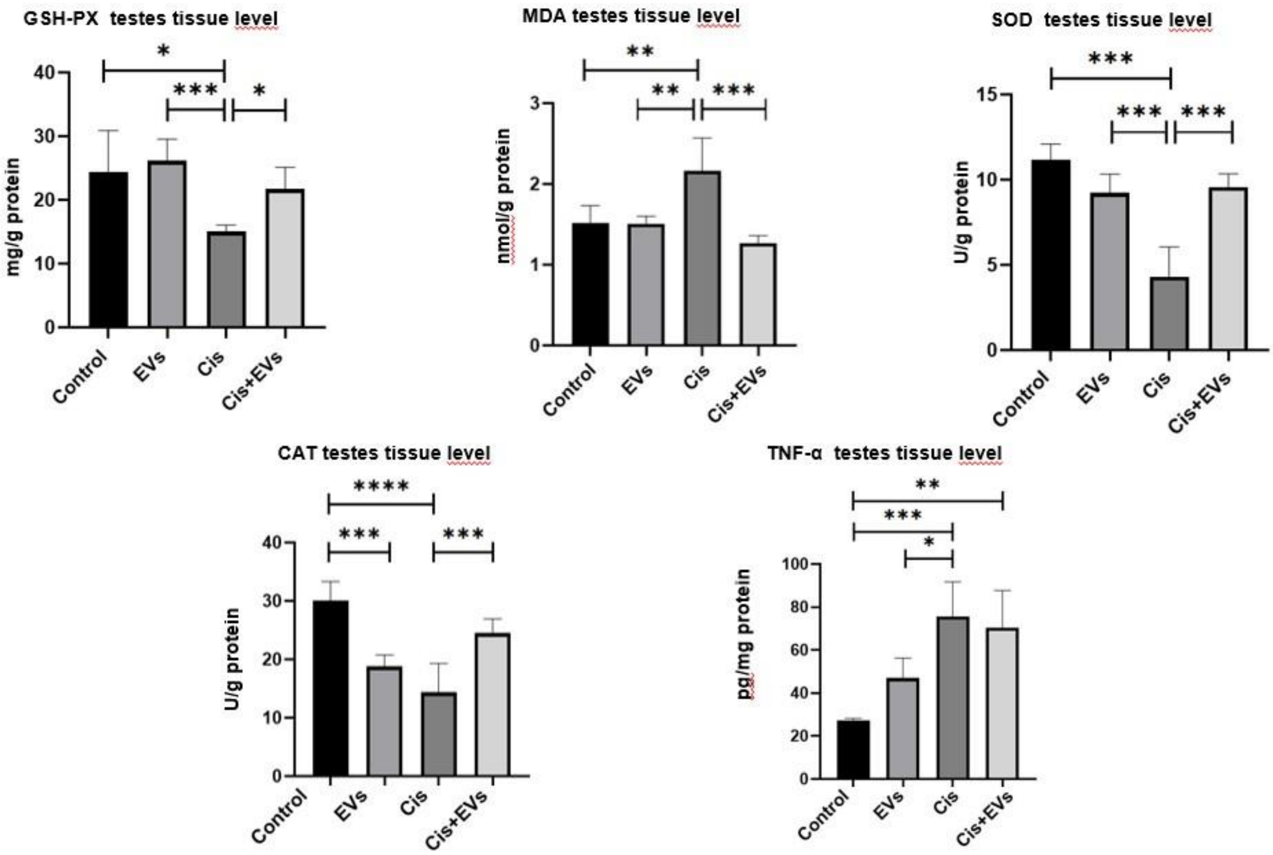

Fig. 7 Statistical analysis of testicular oxidative stress The effects EVs on oxidative stress parameters and apoptotic marker in cisplatin-induced testis damaged in rats. MDA, SOD, CAT, GSH-Px and TNF-α testes tissue level of the experimental groups. ; *, p<0.05, **; p<0.01, ***; p<0.001

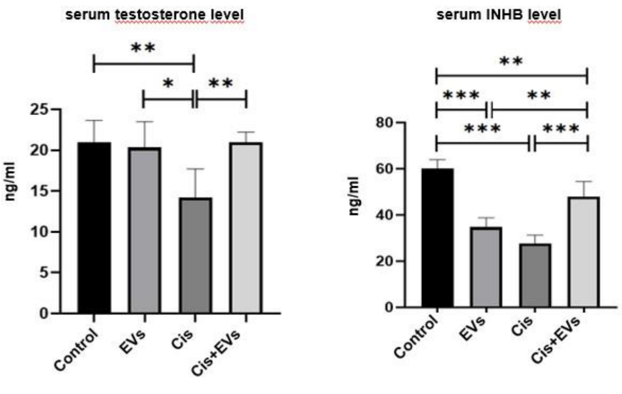

Fig. 8 Statistical analysis of serum hormone levels Serum testesteron and serum INHB level of the experimental groups. ; *, p<0.05, **; p<0.01, ***; p<0.001
