# Supplementary material for: Extracellular vesicles therapy alleviates cisplatin-ınduced testicular tissue toxicity in a rat model
Source: PLoS One. 2025 May 2;20(5):e0314093. doi: 10.1371/journal.pone.0314093 (PMC12047789; doi:10.1371/journal.pone.0314093)
Supplement: Attachment — Submitted filename: Cis, EVs, Testicular toxicity Yildiz.docx [file pone.0314093.s002.docx]

Hi Dr Yildiz

I have gone through your work and want to commend you for the effort which you have made to put this together, especially seeing that this project is likely self-funded. However, I have the following comments to make this better for the entire scientific community;

1. The use of English in this draft does not represent the best scientific expertise expected for a paper like this.

2. From the opening sentence of your abstract , you failed to ignite my enthusiasm about this project. The significance of cisplatin...why cisplatin? Justification for the study? The purpose section in your abstract must be rewritten. This manuscript have great potential if you can do that. Then, the methods... Rewrite your methods so we understand your methodology well...length of experiment, animals? How many? You left me clueless about this experiment here! Then, how did you get your data? Laboratory analysis? Statistical tests? You said testicular morphology was examined using H&E?? Really???? Histoarchitecture or morphology ?? Please add the methods you used for the measurement of hormones, sperm parameters. Your methodology is very poor in this abstract in that... You omitted so many details....

3. The results subsection of your abstract is also faultable. I expect that the results will be presented in a logical sense that follows a thorough discussion of effect and mechanism.

For example; first state the effects on structure, Hormone (function), sperm parameters (function) then the mechanisms of these negative or positive changes (oxidative stress, autophagy)

4. The conclusion of the abstract does not show comprehensive snapshot of your results. I guess you were have a wordcount limit for your abstract but you have to do these corrections to put this manuscript in top shape.

5. Your introduction is needs to be overhauled. From the first sentence with reference (Cisplatin I'd a potent....) to " it has been reported" when you actually didn't add those studies that reported. The third sentence on the genotoxic effects of Cis also lacks merit? Formation of primordial follicle is a genotoxic effects?? Are you sure? You indicated that Cis reduces spermatogenesis? How? What sperm parameters? What stage of spermatogenesis?

6. Then you described EVs as if you didn't get the description somewhere, why? This dampens my enthusiasm, honestly! Each factual statement you make should be backed by a reference.

7. The next paragraph is same. So many missing references. Each factual statement should end with a reference.

8. Replace "long-lived" with a more appropriate adjective.

9. I see no need for the "much research has been focused on starvation-induced autophagy..." What are you trying to say? A lot of missing connections in the paragraph.

10. These paragraphs in the introduction do not connect.

11. I was expecting your introduction will shadow this line of thought

Few sentences on general info about cisplatin. Then it's toxicity on general physiology in humans and animal models. Then, the reprotoxic effects. This should be detailed and more of males. After stating the effects, you discuss the mechanisms. Then, the following paragraph will tow...

"On the other hand, EVs are bla bla..." After the general info about EVs you should talk about effects in several reprotoxicity models , preferably cisplatin or anticancer drugs (if available). You should discuss the mechanisms of its effects exhaustively.

11. Be sure to cite similar works https://doi.org/10.1007/s43188-024-00250-3 in your methodology especially for spermatogenesis, hormones, oxidative stress.

12. Your discussion should tow the same line for the presentation of result. Always be sure to first summarize your findings in the first paragraph of discussion, then discuss histology and morphological parameters in the next paragraph. The following paragraph should discuss findings on hormones...

13. Next, spermatogenesis and sperm parameters, then go to the mechanisms in the subsequent paragraph.

14. Your discussion should include strengths and weaknesses of this study. Your methodology is unique and I am sure there are good things and limitations to it.

15. If possible include graphical abstract. This is not compulsory though.
